# Supplementary figures and images for: The senescence-inhibitory p53 isoform Δ133p53α represses the proinflammatory chemokine CXCL10 in progeria model mice and naturally aged mice
Source: bioRxiv. 2026 Jun 22:2026.03.31.715385. Originally published 2026 Apr 2. Preprint. [Version 3] doi: 10.64898/2026.03.31.715385 (PMC13060196; doi:10.64898/2026.03.31.715385)

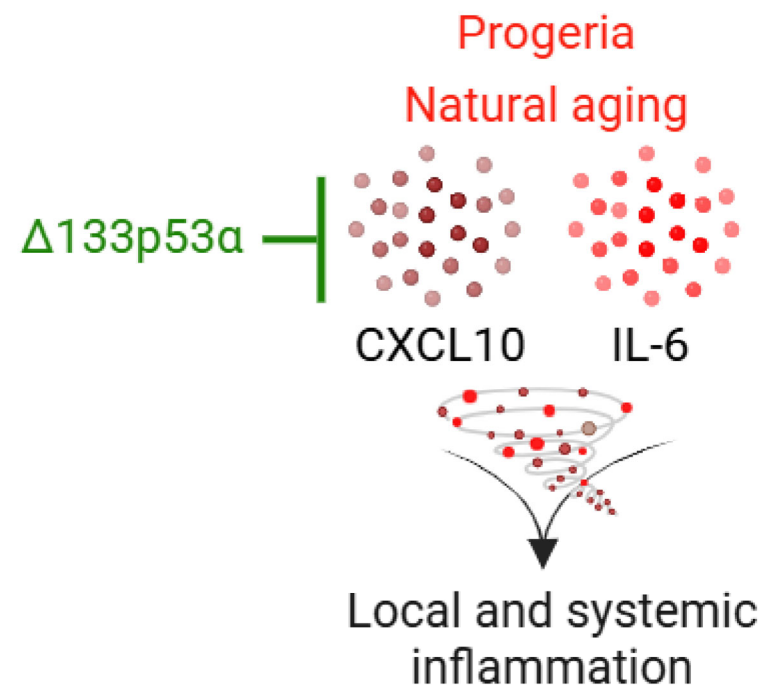

Supplement: Supplement 2 [file media-2.pdf]
