# Supplementary material for: The senescence-inhibitory p53 isoform Δ133p53α represses the proinflammatory chemokine CXCL10 in progeria model mice and naturally aged mice
Source: bioRxiv. 2026 May 5:2026.03.31.715385. Originally published 2026 Apr 2. Preprint. [Version 2] doi: 10.64898/2026.03.31.715385 (PMC13060196; doi:10.64898/2026.03.31.715385)
Supplement: Supplementary file 1 [file media-1.pdf]

Supplementary Figure S1.

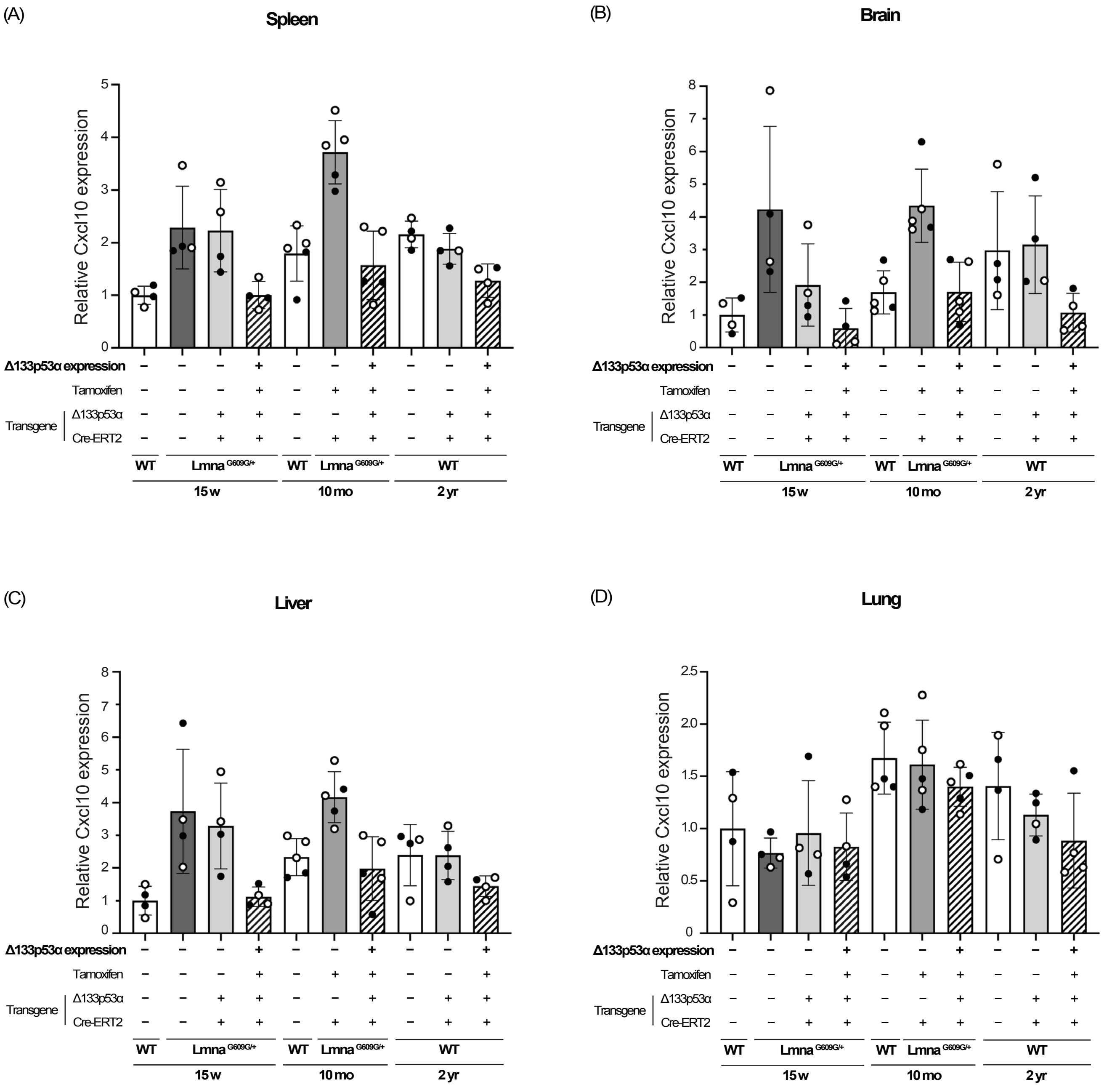

Supplementary Figure S1. All qRT-PCR data in the spleen (A), brain (B), liver (C), and lung (D). Data from *Lmna*$^{G609G/+}$ and wild-type (WT) mice at different ages are shown together for cross-reference. Transgene status and tamoxifen treatment are indicated as in Figures 2 and 3. All data (mean ± s.d.) are presented relative to 15-week-old WT mice (n = 4 or 5; open circles indicate female mice, and closed circles indicate male mice). *P* values are shown in each panel of Figures 2 and 3.
